# Platelet-rich plasma (PRP) in oncological patients: long-term oncological outcome analysis of the treatment of subcutaneous venous access device scars in 89 breast cancer patients

**DOI:** 10.1007/s00404-022-06416-4

**Published:** 2022-04-04

**Authors:** Christian Eichler, Jens Üner, Fabinshy Thangarajah, Julia Radosa, Max Zinser, Lotta Ada Fischer, Julian Puppe, Matthias Warm, Wolfram Malter, Caroline Lenz

**Affiliations:** 1 Breast Cancer Center, St. Franziskus Hospital Muenster, Muenster, Germany; 2 Department of Gynecology and Obstetrics, University of Cologne, Faculty of Medicine and University Hospital Cologne, Cologne, Germany; 3 Department of Radiology, Municipal Hospital Holweide, Cologne, Germany; 4 Department of Gynecology and Obstetrics, Obstetrics and Reproductive Medicine, Saarland University Hospital, Homburg, Germany; 5 Department of Craniomaxillofacial and Plastic Surgery, University of Cologne, Cologne, Germany; 6 Breast Cancer Center, Municipal Hospital Holweide, Cologne, Germany

**Keywords:** Platelet-rich plasma, Breast cancer, Subcutaneous venous access device, Oncological patients

## Abstract

**Purpose:**

Platelet-rich plasma (PRP) is widely used product, and meta-analyses showed this product to be beneficial when applied to a wound area. This study group has already demonstrated increased patient satisfaction and lower complication rates in breast cancer patients who received PRP after removal of their subcutaneous venous access device. This work is a follow-up analysis focusing on oncologic safety. Currently, there is no long-term data on the use of PRP products in cancer patients available yet.

**Methods:**

Between the years 2012–2016, venous access device removal was supported with the application of Arthrex ACP^®^ (Autologous Conditioned Plasma)—a PRP product to improve the wound-healing process. All surgeries were performed in the breast cancer center of the municipal hospital of Cologne, Holweide, Germany. 35 patients received an application of Arthrex ACP^®^ after port removal compared to the control group of 54 patients. Endpoints were local recurrence-free, distant recurrence-free as well as overall survival.

**Results:**

Median follow-up was 45 months. No (0) adverse events were shown for cancer recurrence within the subcutaneous venous access device scar area. Thus, there seems to be no local oncogenic potential of the PRP product. All other endpoints as well as any-cause death numerically favor PRP use.

**Conclusion:**

PRP products such as Arthrex ACP^®^ seem to be oncological inert when applied after removal of subcutaneous access devices. This is the first study providing long-term data about overall survival, distant recurrence-free and local recurrence-free survival after applying PRP in high-risk cancer patients.

## Introduction

Studies have already shown that the application of platelet-rich plasma (PRP) can improve wound-healing processes in orthopedic surgery [1], dermatology [2, 3], ophthalmology [4], gynecology and plastic surgery. Positive results could not only be shown in management of complex wounds but also in conservative PRP treatments of joints [5–7]. Comparative meta-analyses on this topic are available and often compare PRP with corticosteroids for mostly orthopedic procedures [8–10]. The positive effect of PRP on wound healing can primarily be attributed to the included growth factors, such as platelet-derived growth factor (PDGF), basic fibroblast growth factor (bFGF), endothelial growth factor (EGF), transforming growth factor-ß (TGF-ß) and vascular endothelial growth factor (VEGF) [11, 12]. However, the oncological safety of PRP has remained unclear.

Long-term oncological data on any medical product is difficult to obtain. Short-term patient satisfaction after treatment with platelet-rich plasma (PRP) was previously evaluated by our study group. In addition, complication rates and postoperative outcomes were assessed [13].

Complication rates, as well as oncological short-term follow-up after the application of PRP in sentinel node biopsies in breast cancer patients were examined in a previous of our studies. No increased rate of local recurrence was observed during the 30-month follow-up. Lower complication rates were registered after the use of ACP^®^ (Autologous Conditioned Plasma)/PRP compared to the control group, although this difference was not significant [14].

Once chemotherapy is completed, patients may focus on their physical appearance and self-perception and, therefore, consider surgical removal of their port system (venous access device) [15]. While this procedure rarely involves clinical complications, patients still mention unsightly scarring, general discomfort in the surgical area, and occasionally arm pain.

It was the aim of this study to prove oncological safety of platelet-rich plasma providing this follow-up analysis [13].

The individual patient’s perception inevitably influences the evaluation of each product. The mere knowledge of having received a potentially positively effective product affects patients to assess the product positively. A simple evaluation of PRP by questionnaire would be insufficient and potentially biased. To overcome this bias, only objective parameters were assessed in the study presented.

The following questions were asked:

1. Is ACP^®^/PRP oncological inert?
2. Is there a disadvantage in overall survival, distant recurrence-free and local recurrence-free survival after applying ACP^®^/PRP?

## Patients and methods

The study was performed retrospectively at the Municipal Hospital Holweide, Breast Cancer Center, Cologne, Germany. Patients undergoing a removal of a port system (venous access device) between 2012 and 2016 were offered to support this procedure with the application of ACP^®^ (double syringe system). This PRP product is an autologous conditioned plasma system (ACP^®^) by Arthrex^®^. There are different types of PRP products available. The difference is mainly in the method of preparation. This is mostly manufacturer specific. PRP and ACP^®^ are used homologous in this paper. All patients received the ACP^®^/PRP application free of charge.

The ACP^®^/PRP cohort included 35 patients, while the control cohort (no ACP^®^/PRP) consisted of a total of 54 patients. The patients’ ages did not vary between the PRP and the control group. All patients had previously received chemotherapy for early breast cancer. None of the patients underwent radiation treatment on the ipsilateral side. All patient’s nodal status was clinically negative (cN0). As to the nodal status for both subgroups, we refer to previously published paper by this study group [13]. Table 1 shows tumor biology for both groups.

**Table 1:** Tumor biology for both groups

	ACP^®^ and port	%	Port without ACP^®^	%
Gender (w)	35	100.0	54	100.0
Median age (range)	51 (35–74)		58 (37–86)	
Histology				
NST	30	85.7	47	87.0
Lobular	3	8.6	5	9.3
NST/lobular	0	0.0	1	1.9
OTH	2	5.7	0	0.0
DCIS	0	0.0	1	1.9
Grading				
G1	0	0.0	2	3.7
G2	18	51.4	24	44.4
G3	17	48.6	28	51.9
Hormones				
ER positive	22	62.9	39	72.2
ER negative	13	37.1	15	27.8
PR positive	21	60.0	34	63.0
PR negative	14	40.0	20	37.0
HER2/neu				
Positive	5	14.3	7	13.0
Negative	30	85.7	47	87.0
Ki-67 (%)				
< 14	5	14.3	6	11.1
> 14	6	17.1	16	29.6
> 25	23	65.7	31	57.4
No data (DCIS)	1	2.9	1	1.9

(HER2/neu positive = 3+, 2+, FISH positive; HER2/neu negative = 0, 1+)

**Fig. 1:**
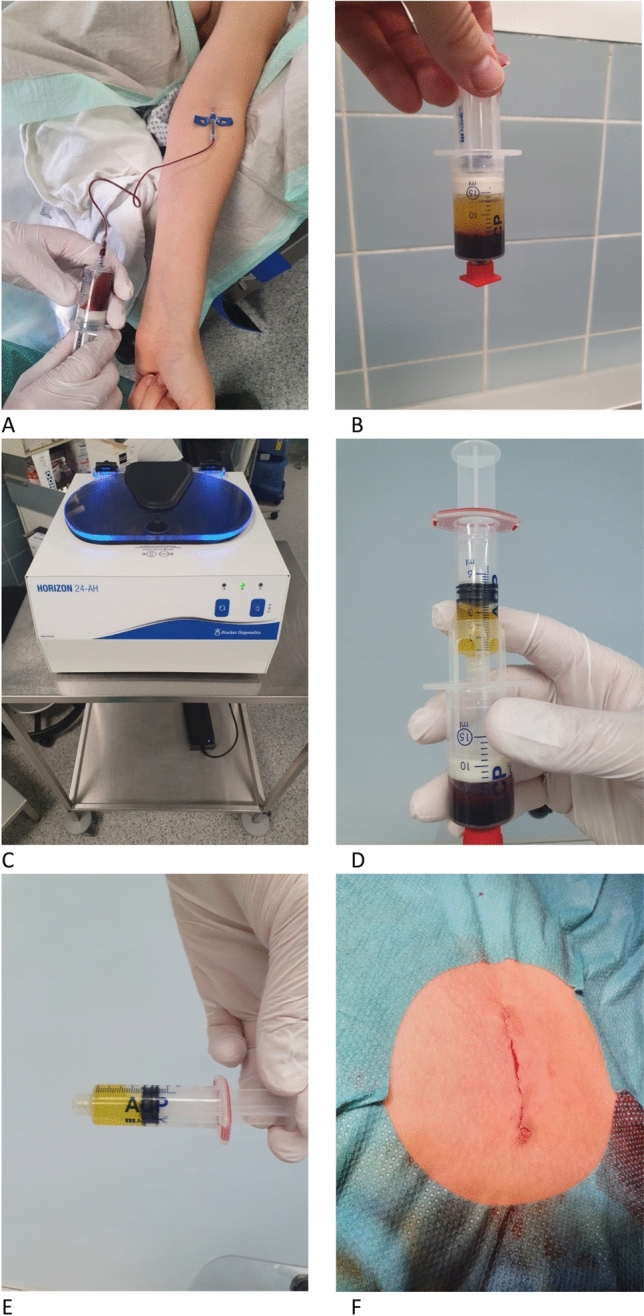
PRP preparation and application. Preparation steps for the Arthrex ACP double syringe, PRP extraction system. **A** Showing the extraction of autologous blood intraoperatively. **B** The separation of PRP and erythrocyte layers after centrifugation (**C**). **D**, **E** The harvesting of PRP via the double syringe system. **E** Wound area after wound closure and introduction of PRP

The ACP^®^ double syringe system (Arthrex, Naples, Florida, USA) was used. The PRP product was obtained and prepared according to the manufacturer’s instructions (Fig. 1). After extraction of patient blood during surgery via port or puncture of a peripheral vein, the blood sample was centrifuged. Injection of PRP was applied subcutaneously under sterile conditions after removal of the port system and wound closure. A detailed description is also given in our previous publication [13]. All surgeries were performed by qualified breast surgeons and both groups underwent the same surgical procedure.

As choosing between injection and no injection already implies a strong bias, only objective evaluations were included in this study. Endpoints were overall survival, distant recurrence-free and local recurrence-free survival as well as any-cause death.

Statistical analysis was performed using SPSS software. Kaplan–Meier plots were used to evaluate overall survival, distant recurrence-free as well as local recurrence-free survival.

Written informed consent has been obtained from each participating patient. A copy is available for review. This study was performed in accordance with institutional review board standard operating procedures. The usage of the patient blood products has been reported to the responsible municipal health agency (i.e., Bezirksregierung Koeln, Dezernat 24: Oeffentliche Gesundheit, medizinische und pharmazeutische Angelegenheiten). An approval/vote was granted by the Ethics Committee at the University of Cologne, Cologne Germany ethics case number #20-1058.

## Results

This is the first long-term follow-up analysis of any kind for ACP^®^/PRP application in a cohort of high-risk cancer patients.

No patients were excluded from this consecutive, retrospective analysis. The median follow-up time was 45 months. Shortest follow-up at time of analysis was 2 months. Even the shortest follow-up of 2 months is at the time of publication at least 23 months. There is zero oncological event recognized for this group as well. Longest follow-up time was 78 months at time of analysis. No (0) cancer recurrence was found within the subcutaneous venous access device scar area.

Mean estimated local recurrence-free survival for patients with ACP^®^ was 72.6 months.

Mean estimated local recurrence-free survival for patients without ACP^®^ 74.1 months. *p *= 0.893 (Table 2).

**Table 2:**
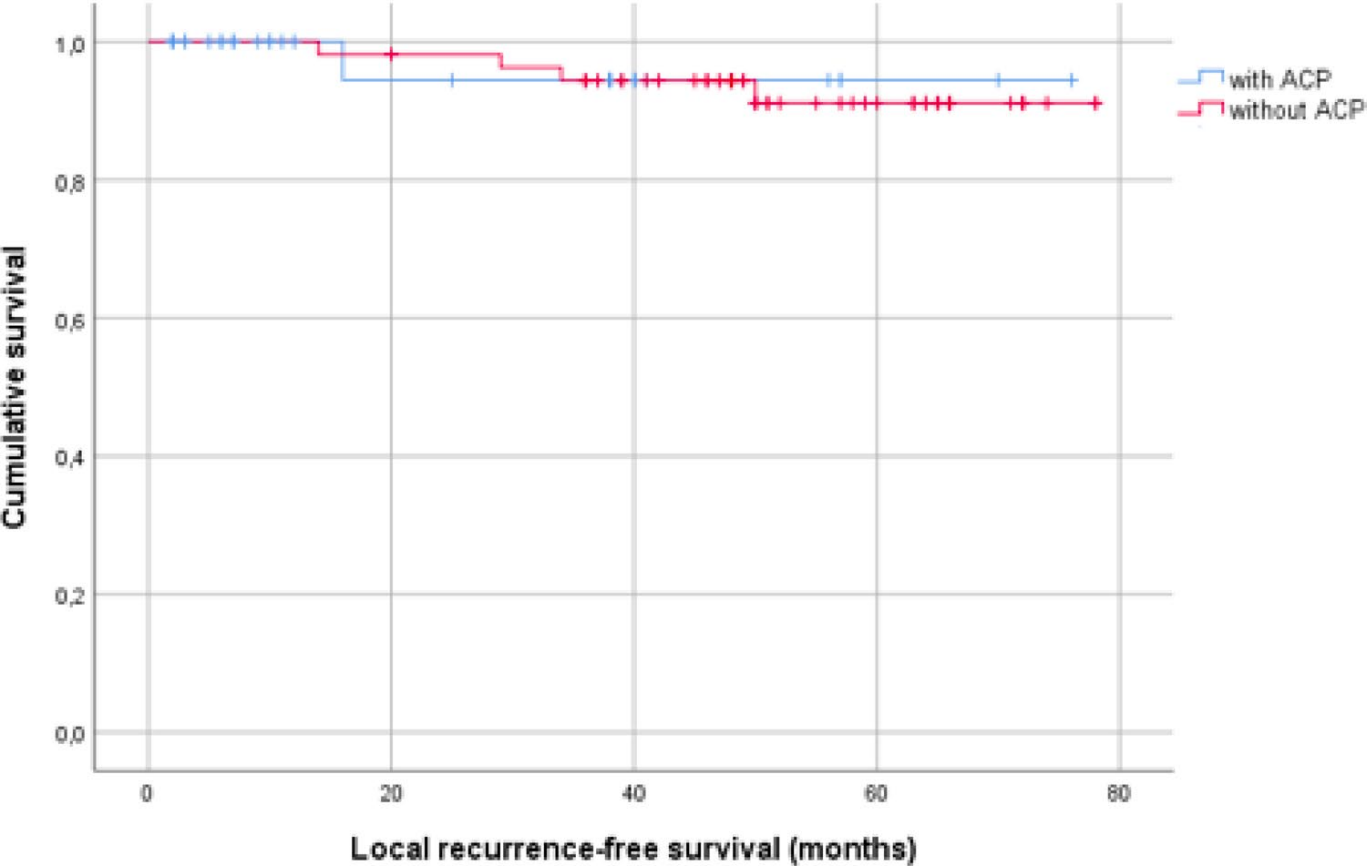
Local recurrence-free survival

Kaplan–Meier plot for local recurrence-free survival of patients treated with or without ACP^®^

Mean estimated distant recurrence-free survival for patients with ACP^®^ was 70.5 months. Mean estimated distant recurrence-free survival for patients without ACP^®^ was 73.5 months. *p *= 0.819 (Table 3).

**Table 3:**
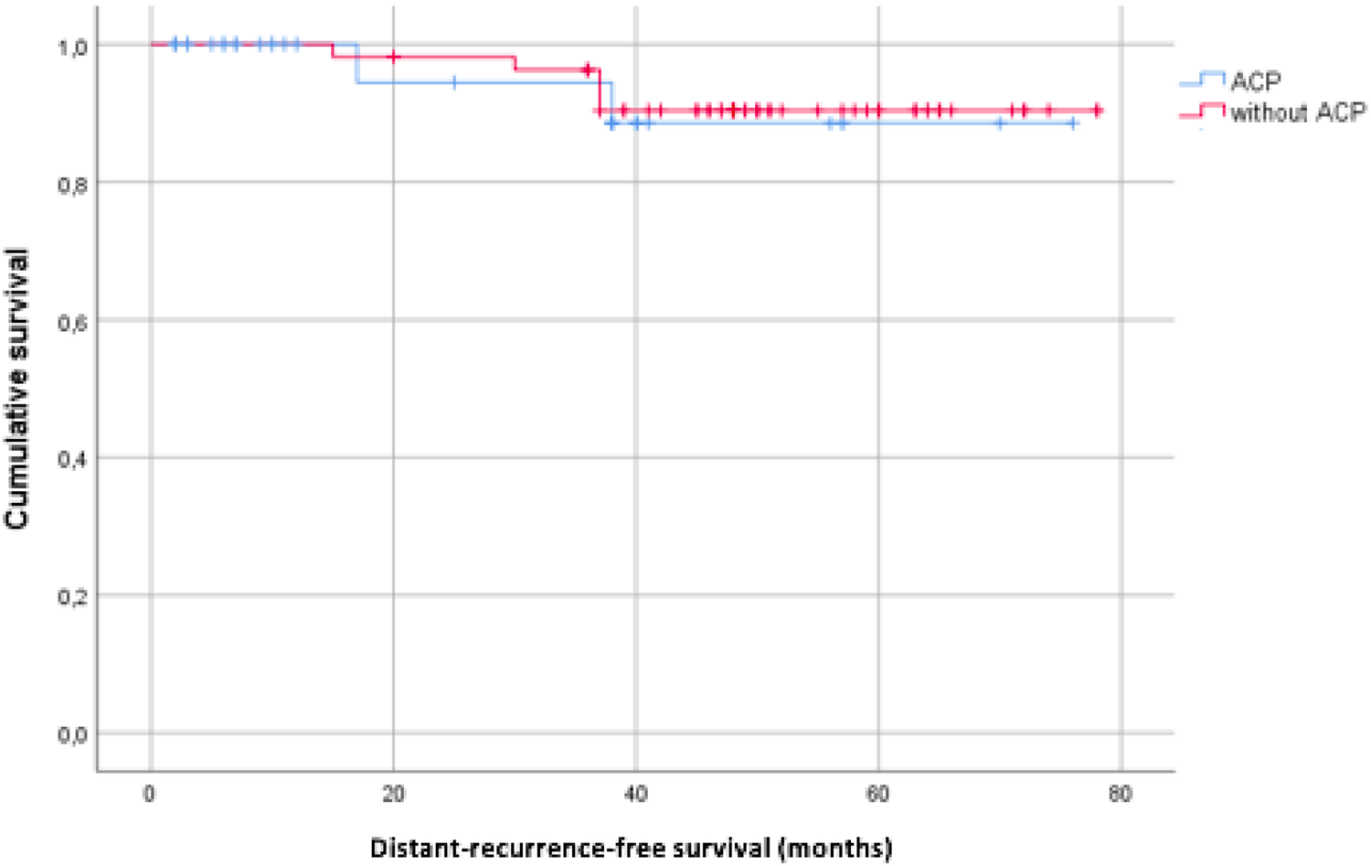
Distant recurrence-free survival

Kaplan–Meier plot for distant recurrence-free survival of patients treated with or without ACP^®^

No deaths were recorded in the group with ACP^®^, four in the group without ACP^®^. Although this result represents a numerical advantage for the ACP^®^ group regarding overall survival, it should be considered as coincidence (Table 4).

**Table 4:**
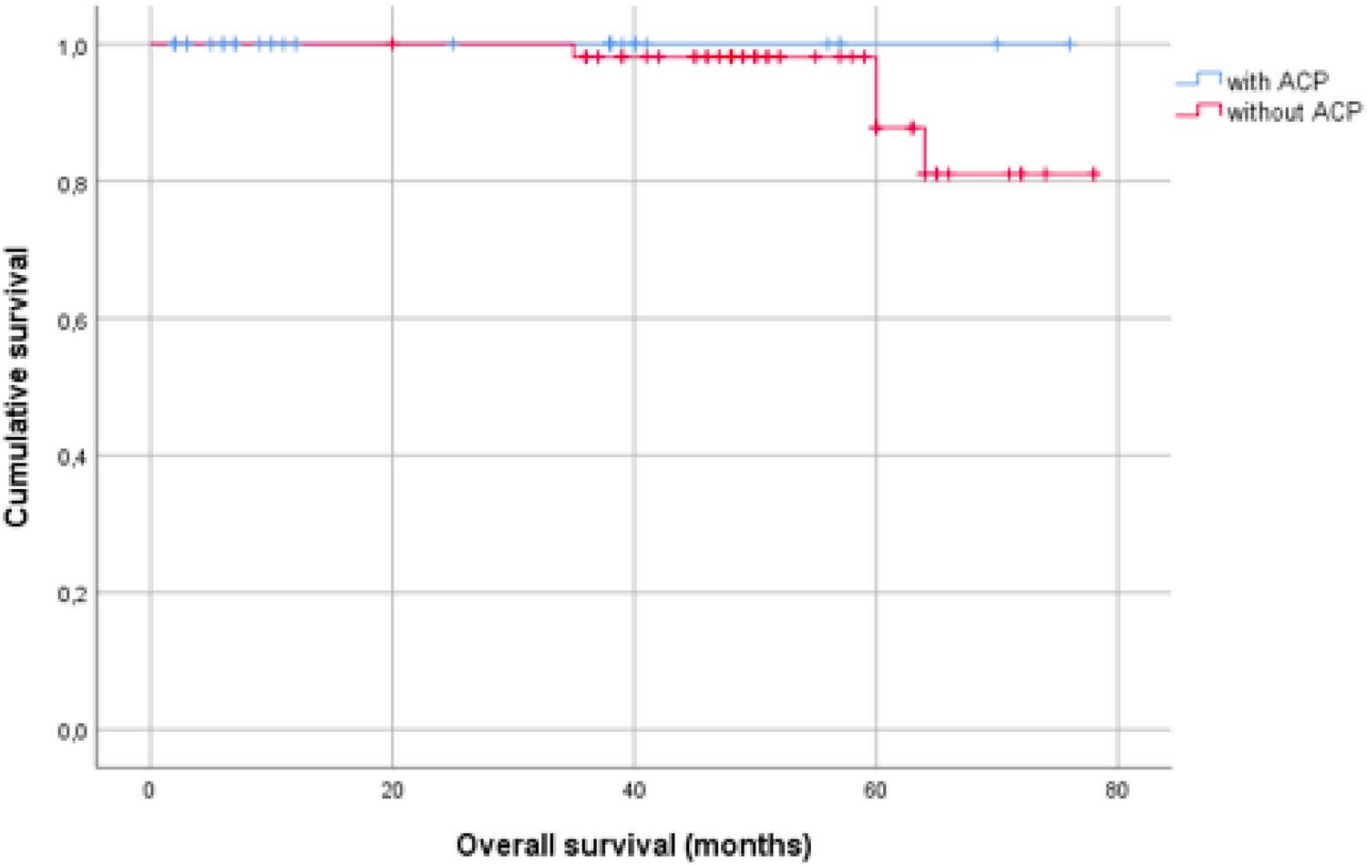
Overall survival

Kaplan–Meier plot for overall survival of patients treated with or without ACP^®^

Overall, oncological safety was given for all patients. PRP does not seem to have a negative oncological impact.

## Discussion

The literature shows PRP to improve the outcome in orthopedics, dermatology, ophthalmology as well as in plastic surgery. Given the fact that PRP contains diverse endogenous growth factors, a review of the oncogenic potential of this product remained essential.

A similar question was already investigated for the use of lipofilling. The literature provides insufficient and contradictory data to demonstrate the safety of lipofilling after breast conserving surgery [16]. Further studies are necessary to evaluate the oncological safety of lipofilling.

Our purpose was, therefore, to investigate the oncological potential of PRP.

Previously, we illustrated significantly improved short-term patient satisfaction, postsurgical outcome and complication rates in breast cancer patients when treated with PRP after removal of their subcutaneous venous access device [13]. Accordingly, we evaluated PRP in sentinel node biopsy procedures for breast cancer patients in terms of complication rates and oncological short-term follow-up. No increase in local recurrence rates and a decrease in complication rates were documented [14].

We assumed PRP to show no long-term effect but currently no long-term data were available on PRP products in any oncological patients yet. This work is a follow-up analysis focusing on oncological safety in a group of high-risk cancer patients. This is the first long-term follow-up analysis for any ACP^®^/PRP data in oncological, specifically breast cancer, patients.

Overall, we found ACP^®^/PRP to have no disadvantages when applied after removal of venous access devices. A numerical advantage regarding overall survival was shown when applying PRP. However, this effect is considered by the authors to be coincidental and does not appear to be due to the application of PRP. Thus, there seems to be no local oncogenic potential of the PRP product when injected to venous port sites. PRP products such as Arthrex ACP^®^ appear to be safe to use for venous port site injections in high-risk cancer patients. The high patient satisfaction should be weighed against the low cost of $50 for each ACP^®^ double syringe system. It is important to consider that improving quality of life may counteract cancer-related cognitive impairment for cancer survivors [17, 18]. The use of PRP should at least be considered and offered to patients.

Limitation to this study to make a final statement about oncological outcome might be a relatively short median follow-up time of 45 months and a small cohort of patients.

There is an ongoing database to evaluate a significant long-term oncological outcome and a larger cohort study is planned.

1. Is ACP^®^/PRP oncological inert?
  Yes. We were able to show follow-up data for ACP^®^/PRP use in high-risk cancer patients. ACP^®^/PRP has not shown any negative side effects. PRP is considered to be reasonable safe when used after port system removal.
2. Is there a disadvantage in overall survival (DS), distant recurrence-free (DFS) and local recurrence-free survival (LFS) after applying ACP^®^/PRP?

OS, DFS und LFS data showed no disadvantage in introducing this growth factor-enriched material (PRP) into wound areas of high-risk oncological patients.

## Conclusion

PRP products such as Arthrex ACP^®^ seem to be safe to use in high-risk cancer patients when applied to venous port sites after removal. Previous studies showed significant advantages for the use of PRP for example, faster wound-healing time and, therefore, reduced length of hospital stay. Wound healing disturbance was less when using PRP [6, 19]. This leads to a higher patient satisfaction. In addition, a relative absence of significant demonstrable adverse effects was observed. Theoretically, possible local infections can be mentioned as negative effects due to the intervention. These could not be detected in our cohort. To date, these are the only available data evaluating oncological safety of PRP. With increased patient satisfaction and presented oncological safety, patients should be offered the application of ACP^®^—also considering the negligible costs.

## Data Availability

Not applicable.
